# Medical and Philosophical News

**Published:** 1781-03

**Authors:** 


In a late *Journal de Medecine*, M. Baillet, furgeon to the Hotel Dieu at St. Vallery fur Somme, gives an account of an extraordinary mifplacement of the ftomach and organs of generation in a fœtus that died a few minutes after its birth. At firft view the moft fingular circumftances attending it feemed to be its having no anus, nor any appearance like the organs of generation, excepting a fmall hole, large enough to admit a probe; every where elfe the fkin had the fame uniform appearance as upon the back and abdomen. Under the fkin where Mr. Baillet obferved this perforation, the fat was an inch and a half in thicknefs. Our author, who had ventured to pronounce the child a female, was not a little furprized, on tracing this paffage with his knife, to find that it terminated in a penis, which, with the reft of the male organs of generation, were concealed under this mafs of fat.

Nothing remarkable appeared in the thorax of this child, but on examining the lower belly M. Baillet found that the œfophagus, inftead of terminating in the ftomach, was connected with the large inteftines; after thefe followed the fmall inteftines, and then the ftomach, which was found in in the place where the rectum ought to have been, and adhering by the cardia to the coccyx.

